# Household Food Insecurity Predicts Childhood Undernutrition: A Cross-Sectional Study in West Oromia (Ethiopia)

**DOI:** 10.1155/2020/5871980

**Published:** 2020-03-09

**Authors:** Wondu Garoma Berra

**Affiliations:** ^1^Department of Nutrition & Food Hygiene, Hubei Key Laboratory of Food Nutrition and Safety, MOE Key Laboratory of Environment and Health, School of Public Health, Tongji Medical College, Huazhong University of Science & Technology, 13 Hangkong Road, Wuhan 430030, Hubei, China; ^2^Wollega University, Nutrition Unit, P.O.Box 395, Nekemte, Ethiopia

## Abstract

**Background:**

Despite mixed reports, food insecurity emerges as a predictor of nutritional status, assumably limiting the quantity and quality of dietary intake. In Ethiopia, the prevalence of childhood undernutrition and food insecurity is highly pronounced. However, whether household food insecurity predicts undernutrition in children was not yet well established. Thus, the aim of the present study was to identify the link between household food access and undernutrition in children aged 6–23 months in West Oromia zones, Ethiopia.

**Methods:**

A cross-sectional study was conducted on a sample of 525 households during June–October 2016. Food access was measured as Household Food Insecurity Access Scale. Semistructured interviewer-administered questionnaires were employed to collect data on sociodemographics, child health, child dietary practices, household food security, and anthropometrics. The height and weight of children aged 6–23 months in each household were measured. Multivariate logistic regression models were constructed to assess the association between household food insecurity and child nutritional status measured from undernutrition indicators.

**Results:**

Overall, more than two-thirds (69%) of households were classified as food insecure (had insufficient access to adequate food), with a mean (SD) household food access score of 7.9 (7.7). The respective prevalence of mild and moderate food-insecure households was 56.6% and 12.4%. Higher proportions of children in food-insecure households were stunted (41.8% vs. 15.5%), underweight (22.0% vs. 6.1%), and wasted (14.9% vs. 6.1%). Overall, the prevalence of child undernutrition was 21.3% in the target population, with 16.2% stunted, 6.9% underweight, and 6.3% wasted. The present finding shows food-secure households were 54% protective (OR: 0.46, 95% CI: 0.25–0.84) for child undernutrition. Compared to children in food-secure households, children who were reportedly living in moderately food-insecure households were over twice more likely stunted (OR: 2.09, 95% CI: 1.02–4.28) and over 4 times more likely underweight (OR: 4.73, 95% CI: 1.81–12.35). However, household food insecurity was not a correlate for acute malnutrition (wasting) in children.

**Conclusions:**

The prevalence of household's food insecurity situation is very common and more pronounced among households with undernourished children aged 6–23 months in Ethiopia. The analysis of this work shows that moderately food-insecure households are a salient predictor for composite undernutrition, stunting, and underweight, but not for wasting. Thus, this finding informs the need for multisectoral strategies and policies to combat household's food insecurity and multiple forms of child undernutrition, beyond the socioeconomic wellbeing.

## 1. Introduction

Complementary feeding is defined as the process starting when breast milk alone is no longer sufficient to meet the nutritional requirements of infants, and therefore, other foods and liquids are needed, along with breast milk. Such transition from breastfeeding to family foods (complementary feeding) typically covers the period from 6 to 24 months of age and is often considered as a critical period of growth during which nutrient deficiencies and illnesses contribute globally to higher rates of undernutrition among children [1, 2].

WHO recommends [2] complementary feeding should be *timely* (meaning all infants should start receiving food in addition to breast milk at 6 months); *adequate* (given in amounts, frequency, consistency, and using a variety of foods, while maintaining breastfeeding); *safe* (prepared and given in a safe manner, to minimize the risk of contamination); and *appropriate* (appropriate texture for the age) and applying responsive feeding following the principles of psychosocial care.

In Ethiopia, inappropriate child feeding practices are mostly attributed to poor dietary quality or poor feeding practice, if not both [3], and are often the main determinants of inadequate intakes than the availability of foods in the households [1, 2]. A bulk of the empirical literature also links inappropriate Infant and Young Child Feeding (IYCF) practices with the high prevalence of childhood undernutrition in many low-income countries [4, 5], which also embraces Ethiopia [6, 7].

As a result, the burden of child undernutrition remains unacceptably high in developing countries, particularly in sub-Saharan Africa (SSA). Overall, one-third of all undernourished children globally reside in sub-Saharan Africa (SSA), with stunting 38.5%, underweight 25%, and wasting 9% [8].

Towards the conclusion of the Millennium Development Goal (MDG), by the target date of 2015, then replaced by the Sustainable Development Goals (SDGs), there was an overall improvement in the prevalence of stunting and underweight for under-five children in Ethiopia [9, 10]. For example, stunting was 58% in 2000, 51% in 2005, 44% in 2011 [9], and 38% in 2015 [10]. The decline in the proportion of stunting among Ethiopian children shows improvement in chronic malnutrition over the past 11 years or more. And also, a similar pattern was observed for underweight, which dropped from 41% in 2000 to 33% in 2005 and 29% in 2011 [9] and then to 24% in 2015 [10]. However, the prevalence of wasting in children in Ethiopia has remained stagnant, 12% in 2000 [9] and 10% in 2015 [10].

Despite discrepancies by geographic regions, Ethiopia has one of the highest rates of childhood undernutrition in the world [11], reportedly with rural areas more malnourished than urban [12]. Concomitantly, poverty and food insecurity are also a widespread and severe problem in Ethiopia [13, 14]. Even, the incidence of food insecurity is severe in western parts, some southern parts, and drought-prone areas of the country [14]. A study conducted by Bealu et al. [11] suggests that even though the role is not clear, food insecurity is considered as one of the determinant factors of malnutrition in developing countries [11]. But it might be assumed that food insecurity affects the nutritional and health status of household members through limiting the quantity and quality of dietary intake.

However, at present, there is a mixed report on the association of household food insecurity and childhood undernutrition. To our knowledge, no comprehensive studies have been reported on the relation between household food insecurity and nutritional status of children in Ethiopia.

Therefore, the aim of this study was to determine the associations between the household's food insecurity and childhood undernutrition in the study setting. In addition, the present study evaluates the prevalence of stunting, underweight, and wasting in targeted children. The primary assumption is that the lack of household's food security is related to nutritional outcome measures.

## 2. Materials and Methods

### 2.1. Study Setting and Study Design

To carry out the study, urban and periurban of Nekemte were purposively selected considering the ecological and geographic areas, so that the current agricultural production, dietary practices, and food security situations are similar across [15, 16] and would be generalizable for the study setting.

In every municipality of Nekemte, the first households were chosen randomly and the subsequent households were visited using systematic method taking into account the estimated total number of households in the cluster. Proportionally, ≥40 households were surveyed in each of the six municipalities. Accordingly, every eligible household was surveyed until the minimum sample sizes for each municipality were achieved. If a participant (mother of a child aged 6–23 months) was not available in her home, repeated visit was conducted later in the day. However, if the participant was not available by the end of the day, a replacement was made from the remaining available households in the respective municipality.

After randomly selecting the potential households of participating families, the study team jointly with health extension worker (HEW) visited them at their homes. With the help of HEW who have full trust of the local people, our study team was introduced to the potential participating family, and the purpose of the visit was explained. After a potential participating family verbally accepted the screening process, the eligibility of the participants was assessed and screened. And then informed consent was also obtained before the study participants were interviewed.

The eligibility requirements of participants to be included for data collection were as follows: (a) households with children aged 6–23 months, who were apparently healthy and not suffering from any disability (such as child born with congenital malformation or other physical/mental disability or child experiencing a severe illness), and (b) mothers/caregivers who were at least 18 years of age at the time of contact, residents of West Oromia for the past 6 months, and willing to participate in the study.

However, as obtaining full records of eligible children living in the study area was difficult, a full support from health extension workers (HEWs) was used to carry out the household survey.

Four data collectors with experience in conducting nutrition and health national surveys were recruited and trained (by principal investigator) for two days on the specific survey objective, methodology/procedures, and review of survey forms question by question including anthropometrics measurements (height and weight), feeding practices, and assessment of household's food security. This training on the objective and procedure of the survey was also given to the participating HEWs who were selected based on their representativeness of each municipality to jointly work with our data collectors.

The present study employed a cross-sectional study design. It involved a household survey to collect information related to household's food security, child's dietary practices, and anthropometric measurements during June–October 2016.

A total of 525 households and young children aged 6–23 months were occupied and interviewed. The calculated sample size was based on the indicator prevalence of 50% (95% CI, ±5 CI width) and 10% attrition, and also considered the large population size in the study setting as suggested by experienced researchers [17]. Accordingly, over 428 sample households were required.

However, as obtaining full records of eligible children in the study area was difficult, we randomly raised the final sample size to 532, of which 525 were occupied and interviewed. This was aimed to increase the chance to capture a sufficient proportion of households for the following target groups: (1) infants aged 6–11 months and (2) children aged 12–23 months.

### 2.2. Data Collection

Semistructured interviewer-administered questionnaires were employed to collect information related to household sociodemographics, household food security, child's health, child feeding practices, and nutritional status of children.

#### 2.2.1. Assessment of Household Food Security

Food security, as defined by the United Nations, means that all people, at all times, have physical, social, and economic access to sufficient, safe, and nutritious food that meets their food preferences and dietary needs for an active and healthy life. An assessment of household's food insecurity was carried out using a standard questionnaire (Household Food Insecurity Access Scale, HFIAS) already developed by Food and Nutrition Technical Assistance II (FANTA) project in collaboration with others [18]. The HFIAS is composed of a set of nine generic questions that have been used in several countries and appear to distinguish food-insecure from food-secure households across different cultural contexts [18]. The information generated by the HFIAS can be used to assess the prevalence of household food insecurity (access component) and to detect changes in the food insecurity situation of a population over time.

In the present study, food insecurity was measured through the Household Hunger Scale (HHS), which focused on lack of food in the household and additional survey questions on any existing anxiety or concerns about accessing sufficient food and affording a diverse diet.

The HHS consists of three occurrence questions and three frequency-of-occurrence questions. First, the HHS occurrence questions ask whether or not a specific condition associated with the experience of food insecurity ever occurred during the previous 4 weeks (as yes or no). Then, if the response is “yes,” HHS frequency-of-occurrence questions ask how often a reported condition occurred during the previous 4 weeks, i.e., as rarely (once or twice), sometimes (three to ten times), or often (more than ten times).

Consequently, a HFIAS score variable is calculated for each household by summing the codes for each frequency-of-occurrence question with precode (0 = no, 1 = rarely, 2 = sometimes, and 3 = often). In this manner, the maximum expected score for a household is 27. The higher the score, the more food insecurity (access) the household experienced; the lower the score, the less food insecurity (access) the household experienced.

Then, the indicator, average Household Food Insecurity Access Scale score, is calculated using the household scores, dividing the sum of HFIAS scores in the sample for the number of HFIAS scores (i.e., households) in the sample.

At the household level, food security refers to the ability of the household to secure, either from its own production or through purchases, adequate food for meeting the dietary needs of all members of the household [18].

#### 2.2.2. Anthropometric Measurements

Anthropometric data were also used to collect information on the current nutritional status of children. Child height to the nearest 0.1 cm and weight to the nearest 0.1 kg were measured. Child height was measured using locally made movable head length boards (in recumbent position). Child weight was measured using calibrated digital balances (AIWA, India) with 100 g precision, with the child minimally clothed.

WHO ProPAN was used to compute nutrition indices (stunting, underweight, and wasting), and the results were classified according to WHO 2006 cutoff points [19].

In this manner, children whose height/length-for-age *Z*-score is below minus two standard deviations (−2SD) from the WHO Child Growth Reference are classified as stunted. Children whose weight-for-age *Z*-scores is below minus two standard deviations (−2SD) from the WHO Child Growth Reference are classified as underweight, whereas children with weight-for-height *Z*-scores below minus two standard deviations (−2SD) from the median of the WHO reference population were considered wasted.

### 2.3. Data Quality

Data quality assurance procedures were started in the field. Completed survey forms were randomly reviewed at spot in the field by supervisor, who was working closely with the field team. And, usually, at the end of the day when the staff returned from the field, the supervisor and principal investigator reviewed all data forms completed during the day to ensure quality data were collected.

And also, data entry and processing were fully handled by principal investigator following standard quality procedures (double entry, use of summary report, checking for outliers, and so on).

### 2.4. Statistical Analysis

SPSS (v21) was used for statistical analysis, and the result was presented as absolute and percentage values for categorical variables.

Our exposure/independent variables were all categorical and include household's hunger (“food-secure household,” “mild food-insecure household,” and “moderate food-insecure household”); child feeding practices (“appropriate feeding practice” and “inappropriate feeding practice”); child health conditions (“healthy” and “sick/unhealthy”); child age (“aged 6–11 months” and “aged 12–23 months”); mothers report of paid work (“yes” and “no”); and mothers education (“yes, attended formal education,” and “no, can't read and write”).

Regarding the association tests between the independent variables and the outcome variables, binary logistic regression model was applied to assess the association between independent variables and child anthropometric measures. Variables that were statistically significant were tested in multiple models and adjusted for age.

In multivariate logistic regression, we estimated models for exposure, or independent variables in relation to suboptimal child growth outcomes, measured from the indicators used to define undernutrition. A backward variable selection method with the likelihood ratio test was used, adopting the model with the highest adjustment according to the Hosmer–Lemeshow test (*p* > 0.05, closer to 1.0).

The analysis and interpretations of data on the prevalence of stunting, underweight, and wasting among children were based on the 2006 WHO Child Growth Standards [19].

#### 2.4.1. Stunting (Height-for-Age)

This index identifies past undernutrition or chronic malnutrition [20]. This term is called length-for-age, for children below 2 years of age. Children whose height-for-age is below minus two standard deviations (−2SD) from the WHO Child Growth Reference are classified as stunted [19].

#### 2.4.2. Wasting (Weight-for-Height)

This index measures body mass in relation to height and reflects current nutritional status [20]. Children with weight-for-height *Z*-scores below minus two standard deviations (−2SD) from the median of the WHO reference population were considered wasted [19].

#### 2.4.3. Underweight (Weight-for-Age)

This index is a composite measure of stunting and wasting and reflects both past (chronic) and/or present (acute) undernutrition [20]. Children whose weight-for-age is below minus two standard deviations (−2SD) from the WHO Child Growth Reference are classified as underweight [19].

Lastly, the prevalence for a composite undernutrition was also calculated from the combination of the three indices above, which we simply termed as undernourished children. This was to represent the prevalence of children who were categorized into one or more indicators (stunting, wasting, and underweight).

Finally, parameter estimates are presented as points (OR) and 95% confidence intervals, considering statistically significant at *p* < 0.05.

## 3. Results

### 3.1. Characteristics of Study Subjects

Table 1 illustrates the general characteristics of the study population. Of the total households successfully covered, 41% were infants aged 6–11 months, and the remaining households (59%) were of older child age-bracket (children 12–23 months).

Nearly, 96% of participating mothers had at least some primary education. Over 92% of participating mothers were reportedly married, believe in Christianity, and were Oromo by ethnicity. This table presents the general description of the study population, including socioeconomy in the study population. For example, 84% of participating mothers had no paid work and they do not participate in any agricultural activities, either. And also, the HFIAS mean and standard deviations were 7.9 (7.7).

### 3.2. Household Hunger and Food Security

Table 2 shows perceptions of household food security and household's hunger situations of the study settings.

Household Hunger Scale (HHS), which focused on lack of food in the household and additional survey questions on any existing anxiety or concerns about accessing sufficient food and affording a diverse diet, was used to measure household's food security situations. Analysis of HHS (%) shows that large proportions of households (56.6%) were categorized into some sort of food insecurity situations, reportedly experiencing little (mild) food insecurity.

As illustrated in Table 2, based on responses to questions in the HHS, about 12.4% households reported experiencing recent hunger—defined by Ballard et al. [21], which is having no food of any kind in the house, members of household going to sleep hungry, or even going no access to food for a whole day and night in the last 30 days preceding the survey.

### 3.3. Nutritional Status, Household's Hunger, and Child Feeding Practices

Table 3 presents cross-tabulation on the prevalence of childhood undernutrition situation, in West Oromia, Ethiopia.

As a whole, the prevalence of child undernutrition was 21.3%, but higher among older children aged 12–23 months. Stunting, underweight, and wasting were 16.2%, 6.9%, and 6.3%, respectively.

### 3.4. Associations for Factors Predicting Undernutrition

Table 4 presents multivariate logistic regression models of household food access situations and undernutrition of children living in the study setting.

As a whole, moderate household food insecurity was found to be the most significant predictor of childhood undernutrition situation in the study setting. Being older children aged 12–23 months and moderate household food insecurity were common risk factors predisposing stunting, underweight, and combined undernutrition among children in West Oromia. Children living in households with moderately food insecure had higher odds for stunting (OR: 2.09, 95% CI: 1.02–4.28) and underweight (OR: 4.73, 95% CI: 1.81–12.35), as compared to those children who were residing in food-secure households. Moreover, children from food-secure households were more likely protected from combined undernutrition (OR: 0.46, 95% CI; 0.25–0.84).

## 4. Discussion

The analysis report of this cross-sectional study has revealed a pronounced prevalence of food-insecure households and undernutrition among children aged 6–23 months in West Oromia, Ethiopia. Also, the present work furthers our understanding and highlights the emerging importance of household food insecurity as the main predictor of undernutrition in children living in limited resource settings.

As a whole, this study reports substantial information that household food insecurity was a significant correlate of composite undernutrition, stunting, and underweight, suggesting that food-secure households may have access to resources that enabled them to overcome frequent, widespread food insecurity that is prevalent in Ethiopia.

Our analysis result shows that the mean of household food access score (mean ± SD) was 7.9 ± 7.7, and the prevalence of food insecurity was 69%. Of which over half (56.6%) of households were categorized into some sort of food insecurity situations, reportedly experiencing little (mild) food insecurity. However, about 12.4% of the households were reported experiencing recent hunger—defined by Ballard et al. [21], which is having no food of any kind in the house, members of household going to sleep hungry, or even going no access to food for a whole day and night during the last 30 days preceding the survey.

Noticeably, high proportion of children associated with food-insecure households were undernourished (54.3% vs. 20.2%), stunted (41.8% vs. 15.3%), underweight (22% vs. 6.1%), and wasted (14.9% vs. 6.1).

Important to note that, however, the overall prevalence of combined undernutrition was 21.3% among children aged 6–23 months in the study population. The stunting rate was 16.2%, which is higher than the other two parameters measured and analyzed (i.e., 6.9% underweight and 6.3% wasted). Although the target age varies, these results were relatively lower than the national (DHS 2016) stunting (38%), underweight (24%), and wasting (10%) reported for under-five children [10].

The most interesting aspect of the present findings was the positive association of moderate household food insecurity with suboptimal child anthropometric measures. This likely suggests that children in the most food-insecure households could have less access to sufficient diet. Our analysis of responses from household food insecurity score reveals that moderately food-insecure households were significantly associated with stunting, underweight, and combined undernutrition in the target children.

The present finding revealed that children who were associated with food-secure households were 54% protected from childhood undernutrition (OR: 0.46, 95% CI: 0.25–0.84), as compared to those children who were living in moderately food-insecure households. Explicitly, moderately food-insecure households were showed a positive correlation with chronic malnutrition (stunting) (OR: 2.09, 95% CI: 1.02–4.28) and underweight (OR: 4.73, 95% CI: 1.81–12.35). However, food-insecure households were not associated with acute malnutrition (wasting).

A similar finding was reported from Ghana, whereby children in food-secure households were 46% protected from chronic malnutrition (OR = 0.54, 95% CI: 0.31–0.94), compared to children in food-insecure households [22]. And also, in consistent with our work, this study reported a strong association of chronic malnutrition (stunting) but not acute malnutrition (wasting) of children to household food access.

Another finding reported from southern regions of Ethiopia by Bealu et al. [11] also confirmed that food insecurity was significantly associated with underweight (AOR = 3.82; CI = 1.78–8.19) and stunting (AOR = 6.7; CI = 3.71–12.1) but not with wasting.

Household's food security is usually considered as a proxy indicator for socioeconomic status of the household [22]. Thus, the pronounced magnitude of household food insecurity recorded in the present study could show the poor food access and socioeconomic status of households in the study area. Despite the national efforts to increase the availability of foods in general, and the presence of diversified agricultural activities in study settings in particular [16], the country still faces serious and growing food insecurity problem, affecting as much as 45% of the population [11]. The present work also supports this notion that a significant proportion of households was experiencing hunger or had anxiety or concerns about accessing sufficient food and affording a diverse diet.

As pointed earlier, evidence from this finding demonstrates that children residing in food-insecure household possibly had limited access to sufficient quality and quantity of food leading to undernutrition, which means that under household food insecure situations, it could be possible that families were unlikely to be able to adopt properly WHO's [23] recommended complementary feeding practices such timely introduction of foods, diet diversity including feeding animal-sourced foods, and continued breastfeeding.

As a whole, the present finding is important that it revealed not only the magnitude of prevalence but also the damaging effect that limited food access could do to the nutritional status of children, even when nutritious foods are locally available, as they may not be accessible to a large percentage of the households. This may be partly due to lack of knowledge on food choice decision making among households in Ethiopia [15], which is also a common problem to many other SSA [19]. Lack of nutrition knowledge for decision making on the selection of locally available nutritious diet and its full cost-benefit analysis could leave households to mostly geared towards abetting hunger as a singularity [24].

At present, there is a mixed report on the association of households hunger and optimal child growth [13, 22, 25–28]. A study reported by Salarkia and colleagues [25] showed no significant association between household food insecurity and mother's feeding status and the occurrence of anemia in the 6- to 24-month-old children.

However, these findings do not rule out the possibility of other micronutrient deficiencies among the food-insecure household children. Similar report was published in Kenya for children under 2 years old [28], which stated a joint influence of food security and wealth status on stunting.

Another interesting finding is a study from Nicaraguans by Schmeer and Piperata [27], which revealed that even mild household food insecurity was reportedly detrimental to child growth during early childhood, further extending into middle childhood ages.

Nevertheless, the present finding reports the importance of household food insecurity likely predisposing childhood undernutrition in limited resource settings. The study would substantially help relevant sectors to strongly give attention and monitor households access to food, not only its magnitude of prevalence, but also the perceived effect of food in secured household on the nutritional status of children. Thus, intervention programs designed to improve child nutritional status in resource-limited settings should consider multidisciplinary approaches engaging nutrition-sensitive interventions to improve household food insecurity, in addition to the socioeconomic wellbeing.

Despite the identified findings, some limitations inherent to our study are acknowledged. Given the scope of the present study, this survey was cross-sectional and it may be dependent on maternal recall which is likely to cause respondent bias. Moreover, data were collected during the rainy preharvest season, and results might not be necessarily extrapolated to other seasons.

## 5. Conclusions

The present findings highlighted on the emerging importance of moderate household hunger as the main predictor of child undernutrition in limited resource settings. Even, moderate food-insecure households were a salient risk factor for child undernutrition all combined, and stunting and underweight in particular, but not for acute malnutrition (wasting). However, these findings do not rule out the possibility of other factors among the food-insecure household children, and these need further investigation.

## Figures and Tables

**Table 1:** Characteristics of the study population.

Population characteristics (*n* = 525)	*n* (%)
Residence (location)	
Urban	294 (56.0)
Periurban	231 (44.0)

Child age category	
Aged 6–11 months	215 (41.0)
Aged 12–23 months	310 (59.0)

Sex of child	
Male	253 (48.2)
Female	272 (51.8)

Mother's/caregiver's age (years)	
18–24 years	219 (41.7)
25–34 years	245 (46.7)
35–49 years	45 (8.6)
≥49 years	2 (0.4)
Missing data	14 (2.7)

Marital status	
Married	494 (94.1)
Single	10 (1.9)
Others (living together)	21 (4.0)

Ethnic group	
Oromo	488 (93.0)
Amhara	21 (4.0)
Others	16 (3.0)

Religion	
Christian	483 (92.0)
Muslim	42 (8.0)

Maternal educational status	
Attended formal education	503 (95.8)
Cannot read and write (did not go to school)	22 (4.2)

Mother's report of paid work	
Yes	84 (16.0)
No	441 (84.0)
Household food access score	**7.9 (7.7)** ^*∗*^

^*∗*^Mean (SD): mean and standard deviations. Values are *n* (%) unless stated

**Table 2:** Perceptions of household food security and household hunger situations of the study settings.

Household food insecurity access conditions	Categories of food insecurity (access) and prevalence, *n* (%)			
	Food secure	Rarely	Sometimes	Frequently
Reports worrying because household would not have enough food, last 30 days	232 (44.2)	45 (8.6)	206 (39.2)	42 (8.0)
Reports having household member had to eat the kinds of foods not preferred because of a lack of resources, last 30 days	225 (42.9)	59 (11.2)	185 (35.2)	56 (10.7)
Reports eating a limited variety of foods due to a lack of resources, last 30 days	241 (45.9)	91 (17.3)	143 (27.2)	50 (9.5)
Reports household member have to eat foods that really did not want to eat because of a lack of resources to obtain other types of food, last 30 days	340 (64.8)	95 (18.1)	83 (15.8)	7 (1.3)
Reports household member had to eat a smaller meal than needed because there was not enough food, last 30 days	318 (60.6)	72 (13.7)	125 (23.8)	10 (1.9)
Reports household member had to eat fewer meals because there was not enough food, last 30 days	433 (82.5)	38 (7.2)	51 (9.7)	3 (0.6)
Reports not having food of any kind in the house because of lack of resources to get food, last 30 days	447 (85.1)	36 (6.9)	42 (8.0)	0 (0.0)
Reports household member going to sleep at night hungry because there was not enough food, last 30 days	489 (93.1)	13 (2.5)	23 (4.4)	0 (0.0)
Reports household member going a whole day and night without eating anything because there was not enough food, last 30 days	526 (98.2)	5 (1.0)	4 (0.8)	0 (0.0)

Household hunger categories and HHS (%)	Food secure (HHS: 0)			31.0%
	Mild food insecurity (HHS: 1)			56.6%
	Moderate food insecurity (HHS: 2–3)			12.4%
	Severe food insecurity (HHS: 4–6)			0.0%

“Rarely” is once or twice in the past 30 days preceding the survey; “sometimes” is 3–10 times in the past 30 days preceding the survey; “frequently/often” is more than 10 times in the past 30 days preceding the survey.

**Table 3:** Prevalence of household food security situation and undernutrition of children in the study setting.

Household food access category	Prevalence, *n* (%)				
	Total, *n* (%)	Stunting	Underweight	Wasting	Undernourished children (composite)^*∗*^
Food secure	163 (31.0)	25 (4.8)	10 (1.9)	10 (1.9)	33 (6.3)
Mild food insecurity	297 (56.6)	42 (8.0)	15 (2.9)	17 (3.2)	56 (10.7)
Moderate food insecurity	65 (12.4)	18 (3.4)	11 (2.1)	6 (1.1)	23 (4.4)
Overall (%)		**16.2**	**6.9**	**6.3**	**21.3**

^*∗*^Undernourished children means those children categorized at least in any of the three indices (i.e., stunted, underweight, and wasted).

**Table 4:** Logistic model examining associations between household food access situations and undernutrition of children in the study setting.

Parameters	Stunting			Underweight			Wasting			Undernourished (combined)^d^		
	*n* (%)	AOR (95% CI)	*p* value	*n* (%)	AOR (95% CI)	*p* value	*n* (%)	AOR (95% CI)	*p* value	*n* (%)	AOR (95% CI)	*p* value
*Household food access category*												
Food secure	25 (4.8)	1		10 (1.9)	1		10 (1.9)	1		33 (6.3)	1	
Mild household food insecurity	42 (8.0)	0.90 (0.52–1.56)	0.71	15 (2.9)	0.77 (0.33–1.78)	0.54	17 (3.2)	0.79 (0.35–1.80)	0.57	56 (10.7)	0.54 (0.28–1.06)	0.08
Moderate household food insecurity	18 (3.4)	2.09 (1.02–4.28)	0.04^a^	11 (2.1)	4.73 (1.81–12.35)	0.002^b^	6 (1.1)	1.42 (0.46–4.36)	0.54	23 (4.4)	0.46 (0.25–0.84)	0.01^e^

*Mothers usual feeding practices*												
Appropriate child feeding practice	69 (13.1)	1		29 (5.5)	1		28 (5.3)	1		91 (17.3)	1	
Inappropriate child feeding practice	16 (3.0)	0.95 (0.51–1.77)	0.89	7 (1.3)	1.16 (0.47–2.87)	0.75	5 (1.0)	0.58 (0.21–1.56)	0.28	21 (4.0)	0.86 (0.49–1.50)	0.60

*Mothers attended formal education*												
Yes, had attended formal education	82 (15.6)	1		32 (6.1)	1		29 (5.5)	1		105 (20.0)	1	
No, can't read and write	3 (0.6)	0.83 (0.23–2.97)	0.78	4 (0.8)	3.94 (1.20–12.86)	0.02^b^	4 (0.8)	3.34 (1.06–10.55)	0.04^c^	7 (1.3)	1.77 (0.68–4.59)	0.24

*Mothers report of paid work status*												
Yes, had paid work	9 (1.7)	1		2 (0.4)	1		2 (0.4)	1		10 (1.9)	1	
No, had no paid work	76 (14.5)	1.57 (0.73–3.37)	0.25	34 (6.5)	2.55 (0.58–11.26)	0.22	31 (5.9)	2.90 (0.68–12.4)	0.15	102 (19.4)	2.06 (1.00–4.23)	0.05^e^

*Child health condition*												
Healthy	71 (13.5)	1		35 (6.7)	1		30 (5.7)	1		95 (18.1)	1	
Sick (recovering from acute illness)	14 (2.7)	1.45 (0.71–2.99)	0.31	1 (0.2)	0.10 (0.01–0.78)	0.03^b^	3 (0.6)	0.76 (0.22–2.62)	0.67	17 (3.2)	1.25 (0.64–2.43)	0.51

*Child age*												
6–11 months of age	12 (2.3)	1		8 (1.5)	1		12 (2.3)	1		24 (4.6)	1	
12–23 month of age	73 (13.9)	5.20 (2.74–9.89)	<0.0001^a^	28 (5.3)	2.48 (1.08–5.68)	0.03^b^	21 (4.0)	1.09 (0.51–2.32)	0.82	88 (16.8)	3.15 (1.92–5.17)	<0.0001^e^

^a^Predictors of stunting—child age and moderate household food insecurity. ^b^Predictors of underweight—child age, maternal education, child health, and moderate household food insecurity. ^c^Predictors for wasting—maternal education. ^e^Predictors for undernourished—child age, maternal work, and moderate household food insecurity. ^d^Undernourished children—means, a composite prevalence of children categorized to one or more indices (stunting, underweight, and wasting).

## Data Availability

The datasets used and/or analyzed during the current study are available from the corresponding author upon request.

## Abbreviations

CI: Confidence interval
DHS: Demographic and Health Survey
HAZ: Height-for-age *Z*-score
HFIAS: Household Food Insecurity Access Scale
HR: Hazard ratio
IYCF: Infant and Young Child Feeding
MDG: Millennium Development Goal
OR: Odds ratio
SAS (software): Statistical Analysis System
SD: Standard deviations
SPSS: Statistical Package for Social Sciences
SSA: Sub-Saharan Africa
UNICEF: United Nation Children's Fund
WAZ: Weight-for-age *Z*-score
WHO: World Health Organization
WHZ: Weight-for-height *Z*-score.
